# Quantifying the Dynamics of Bacterial Biofilm Formation on the Surface of Soft Contact Lens Materials Using Digital Holographic Tomography to Advance Biofilm Research

**DOI:** 10.3390/ijms25052653

**Published:** 2024-02-24

**Authors:** Igor Buzalewicz, Aleksandra Kaczorowska, Wojciech Fijałkowski, Aleksandra Pietrowska, Anna Karolina Matczuk, Halina Podbielska, Alina Wieliczko, Wojciech Witkiewicz, Natalia Jędruchniewicz

**Affiliations:** 1 Department of Biomedical Engineering, Faculty of Fundamental Problems of Technology, Wroclaw University of Science and Technology, 50-370 Wroclaw, Poland; aleksandra.kaczorowska@pwr.edu.pl (A.K.); aleksandra.pietrowska@pwr.edu.pl (A.P.); halina.podbielska@pwr.edu.pl (H.P.); 2 Research and Development Centre, Regional Specialist Hospital in Wroclaw, 73A H. M. Kamienskiego St., 51-124 Wroclaw, Poland; wojciech.witkiewicz@wssk.wroc.pl (W.W.); natalia.jedruchniewicz@wssk.wroc.pl (N.J.); 3 Laboratory of Cytobiochemistry, Faculty of Biotechnology, University of Wroclaw, 14a F. Joliot-Curie St., 50-383 Wroclaw, Poland; 4 LipoTech Ltd., Liszki 536, 32-060 Liszki, Poland; wojtek.fijalkowski@lipid-systems.pl; 5 Department of Pathology, Division of Microbiology, Faculty of Veterinary Medicine, Wroclaw University of Environmental and Life Sciences, 31 C.K. Norwida St., 51-375 Wroclaw, Poland; anna.matczuk@upwr.edu.pl; 6 Department of Epizootiology and Veterinary Administration with Clinic of Infectious Diseases, Wroclaw University of Environmental and Life Sciences, 45 Grunwaldzki Square, 50-366 Wroclaw, Poland; alina.wieliczko@upwr.edu.pl

**Keywords:** digital holographic tomography, quantitative phase imaging, bacterial biofilms, *P. aeruginosa*, *S. epidermidis*, dynamics of biofilm formation, soft contact lenses

## Abstract

The increase in bacterial resistance to antibiotics in recent years demands innovative strategies for the detection and combating of biofilms, which are notoriously resilient. Biofilms, particularly those on contact lenses, can lead to biofilm-related infections (e.g., conjunctivitis and keratitis), posing a significant risk to patients. Non-destructive and non-contact sensing techniques are essential in addressing this threat. Digital holographic tomography emerges as a promising solution. This allows for the 3D reconstruction of the refractive index distribution in biological samples, enabling label-free visualization and the quantitative analysis of biofilms. This tool provides insight into the dynamics of biofilm formation and maturation on the surface of transparent materials. Applying digital holographic tomography for biofilm examination has the potential to advance our ability to combat the antibiotic bacterial resistance crisis. A recent study focused on characterizing biofilm formation and maturation on six soft contact lens materials (three silicone hydrogels, three hydrogels), with a particular emphasis on *Staphylococcus epidermis* and *Pseudomonas aeruginosa*, both common culprits in ocular infections. The results revealed species- and time-dependent variations in the refractive indexes and volumes of biofilms, shedding light on cell dynamics, cell death, and contact lens material-related factors. The use of digital holographic tomography enables the quantitative analysis of biofilm dynamics, providing us with a better understanding and characterization of bacterial biofilms.

## 1. Introduction

The rise of the bacterial resistance to conventional antibiotic therapies signifies that the research on the rapid, reliable, and accurate detection of bacteria, as well as on the examination of the bacterial colonization of different kind of surfaces, are crucial in the fight against bacterial resistance [1]. Particularly important is the colonization of surfaces, as this may lead to the formation of the bacterial biofilm. This is a complex bacterial community, with a highly heterogenous spatial structure of bacteria attached to the surface in self-produced slime, and encased in extracellular polymeric substances, which can be formed at any liquid–solid interface. Moreover, unlike the planktonic bacteria, they can exhibit significantly greater resistance to the commonly used antibiotic, which makes them so dangerous to human health [2]. The biofilms can be formed on surfaces of different materials, starting from those commonly used in the food industry, the kitchen [3,4,5], or the water supply industry [6,7], and ending on surgical instrumentation [8] or medical implants [9].

Bacterial biofilms are also a serious threat in the field of ophthalmology [10,11]. In this case, the risk of biofilm-related infection is associated with the biofilm presence on ocular foreign bodies and used medical devices, or materials relevant to the ocular treatment, such as contact lenses, scleral buckles, suture material, and intraocular lenses [10]. For example, endophthalmitis may be caused by the biofilms of coagulase negative *Staphylococci* and *Propionibacterium acnes*, found on intraocular lenses, keratitis and conjunctivitis by *S. aureus*, *S. epidermidis,* and other *Staphylococcus* species, *Pseudomonas aeruginosa* or *Serratia* spp. biofilms on contact lenses, a scleral buckle infection by Gram-positive cocci biofilms on scleral buckles, or lacrimal system infections by *Staphylococcus* spp., *Pseudomonas aeruginosa*, and *Mycobacterium chelonae* biofilms on nasal intubation devices [12,13,14].

For these reasons, the development of new, non-destructive bacteria detection techniques and procedures is particularly important to characterize the dynamics of biofilm formation directly on the examined surface. The quantitative phase imaging methods, such as digital holographic tomography (DHT) [15,16], seem promising for this area of study. It combines the concepts of digital holographic microscopy [17] and the optical diffraction tomography [18], which enables the reconstruction of the 3D refractive index (RI) distribution of examined samples. The RI is an important biophysical parameter, which is directly related to the density and chemical composition of the examined biological objects. Therefore, based on RI data, it is not only possible to visualize the bacterial biofilms in a truly label-free, non-destructive, and non-contact manner, but also to quantitatively characterize the changes in RI and the volume of the biological samples, which have caused by physiological and pathological processes or induced by external factors. The results reported so far show that DHT can be used to characterize the accumulation of photosensitizers used in antimicrobial photodynamic therapy inside bacterial cells [19,20], as well as to evaluate the effectiveness of bacteria photoinactivation [21], the effectiveness of antibiotic-treatment, and its effect on the RI of bacteria cells [22]. Therefore, DHT may also be valuable in multi-parametric phenotyping the biofilms structures. This has already been verified by our group, since our previously reported study demonstrates that DHT can be used for the detection of biofilm formation on glass surfaces [23]. Unlike other commonly used methods, such as crystal violet staining [24], CFU counting techniques [25], ATP bioluminescence assay [26], and fluorescence microscopy [27], DHT does not require the removal of the biofilm from the surface on which it was formed and does not require any chemical reagents or fluorescent probes for biofilm characterization.

For these reasons, the main objective of this study was to investigate the possible use of DHT for the quantitative, multi-parametric, non-destructive, label-free characterization of bacterial biofilm formation, directly on the surface of soft contact lens materials. The study was carried out on two bacterial species: *Staphylococcus epidermis* and *Pseudomonas aeruginosa*. As mentioned above, these are common causes of biofilm-associated ocular infections. For a comprehensive study, six types of soft contact lens materials were used: three hydrogels and three silicone hydrogels, with different hydration levels, wearing schedules, and oxygen permeability. Based on the reconstructed RI data, it was possible to quantitatively characterize any species- and time-dependent changes in the RI of biofilms, the influence of cell division and cell death on the single-cell’s RI in relation to cell viability, changes in the RI of biofilms in relation to the type of soft contact lens material, and species- and time-dependent changes in the volume of bacterial biofilms formed directly on the studied surfaces. Furthermore, the additional study focused on the possible use of DHT to investigate the effect of the novel liposomal formulation of latanoprost in the form of eye drops on the dynamics of biofilm formation. The studied formulation does not contain any preservatives, therefore, any detailed knowledge on the biofilm formation after application is of high interest. The results obtained confirmed that DHT is a suitable and versatile (label-free, non-destructive, non-contact) technique for the multiparametric, quantitative phenotyping of bacterial biofilms and the dynamics of their formation directly on the surfaces of the soft contact lens materials. According to our knowledge, this is the first attempt to use DHT for the quantitative examination of the dynamics of the biofilm formation of soft contact lens materials directly on the examined surfaces.

## 2. Results and Discussion

### 2.1. Species- and Time-Dependent Changes in the Average RI of Bacterial Biofilms Formed on the Surface of Soft Contact Lens Materials

The refractive index is directly related to the density of the sample. In the case of the RI data provided via DHT, it is possible to perform the spatially resolved examination of the changes of the intracellular composition and density. Any changes in the RI can be related to a local change in cell density, caused by physiological processes (e.g., cell division), cell death, or the penetration of substances, such as photosensitizers, dyes, etc. Therefore, all these factors must be considered if the RI is to be used for the phenotyping of bacteria (single-cells or biofilms).

**Figure 1:**
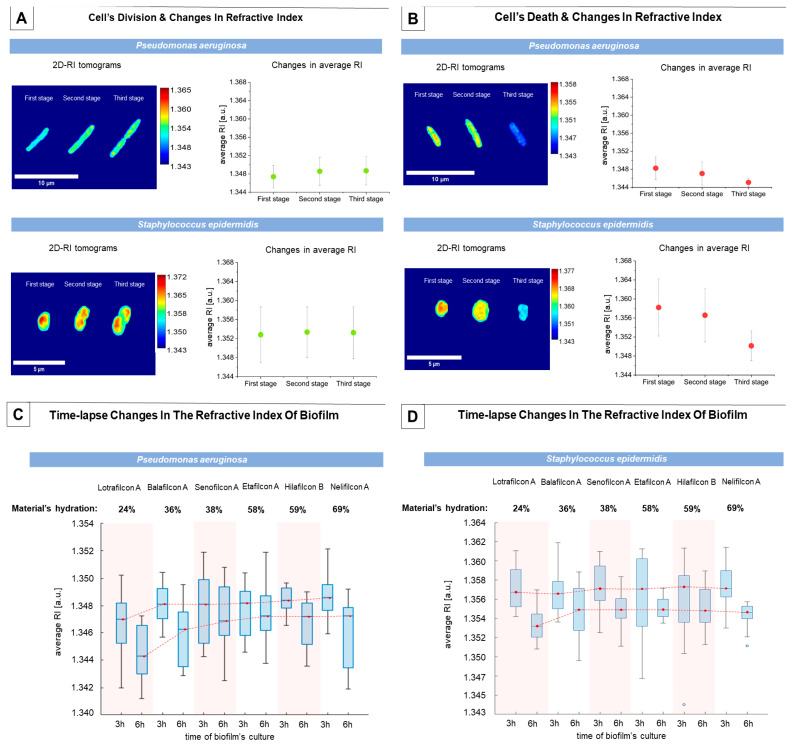
Examinations of changes in the RI of single cells and bacterial biofilms: (**A**) changes in the RI of bacterial cells associated with cell division, (**B**) changes in the RI of bacterial cells associated with cell death, (**C**) boxplot demonstrating the time-lapse changes in the RI of *P. aeruginosa* biofilms formed on the surface of different soft contact lens materials, (**D**) boxplot demonstrating the time-lapse changes in the RI of *S. epidermidis* biofilms formed on the surface of different soft contact lens materials (red lines correspond to the changes of the median value of the average RI).

It should be noted that during the time-lapse examination of such complex structures as biofilms, the different bacterial cells of which it is composed may be at different stages of cell division at the time of measurement; therefore, the variation in the average RI values may be related to the physiological processes taking place in living cells. Therefore, it was decided to perform the initial evaluation of the changes in the RI of single cells caused by the cell division process. Average RI values were calculated from the RI values of the cells in the maximum RI projection: a 2D RI tomogram, corresponding to the central section through the bacterial cell. The results obtained (see Figure 1A) have shown that, during this process, the average RI increases, which is related to the local changes in cell density as the cell elongates (first stage in Figure 1A). Subsequently, the cytoplasm and cell contents move towards the two poles of the cell to form a septum (second stage), ultimately resulting in the division of the original cell into two daughter cells (third stage). It was also shown that the average RI values of *S. epidermidis* cells are significantly higher than those of *P. aeruginosa* cells, which may be related to the smaller size and higher density of these cells.

The bacterial biofilm is a very complex and dynamic biological community, in which there are living and dividing cells, as well as dying cells. During the study, it was also possible to quantitatively analyze the process of the death of single bacterial cells (see Figure 1B). Initially, cells have the same RI values and size as living cells (first stage); however, they then increase in size, leading to a decrease in the cell density and RI (second stage). Finally, the size and RI of the cell decreases (third stage), which is associated with the loss of cell wall integrity. Therefore, the results clearly show that a decrease in the RI over time, below the range of the RI values of living cells, indicates cell death. Our previously reported study showed that the decrease in the RI of *E. coli* cells within the biofilm formed on glass surfaces was related to the decrease in cell viability, which was confirmed by the results of standard comparative microbiological techniques [23]. It was also confirmed by the examination of the bacterial cell’s death, induced by the antibiotic’s treatment [22]. Moreover, a comparison of the results for both species shows that the decrease in the refractive index is much more significant for *S. epidermidis* cells than it is for *P. aeruginosa* cells, which may be related to the different morphological structures and sizes of cells. In addition, the results show that the refractive index, being a fundamental biophysical quantity, can be used as a marker for the viability of the bacterial cells. Based on the average RI values of single cells at stages 1 and 3 of cell death, it was possible to determine the decrease in the RI, i.e., the difference between the average RI values of live/dividing cells and dying cells; this was 4.933 × 10^−3^ RIU (Refractive Index Units) and 9.161 × 10^−3^ RIU for *P. aeruginosa* and *S. epidermidis*, respectively.

As mentioned above, bacterial biofilm is a dynamic community of cells, containing both a population of living or dividing cells and a population of dying cells. This means that the average RI of the entire biofilm depends on which population is predominant. When the subpopulation of living cells predominates, the average RI will fluctuate, but will remain high, whereas, if the subpopulation of dying cells predominates, the average RI of the biofilm will decrease. This indicates that the RI data obtained via DHT may be a valuable marker of the cell’s viability and biofilm maturation. Therefore, taking this consideration into account, a time-lapse was performed, regarding the examination of the average RI values of biofilms formed on the surface of the examined soft contact lens material.

**Figure 2:**
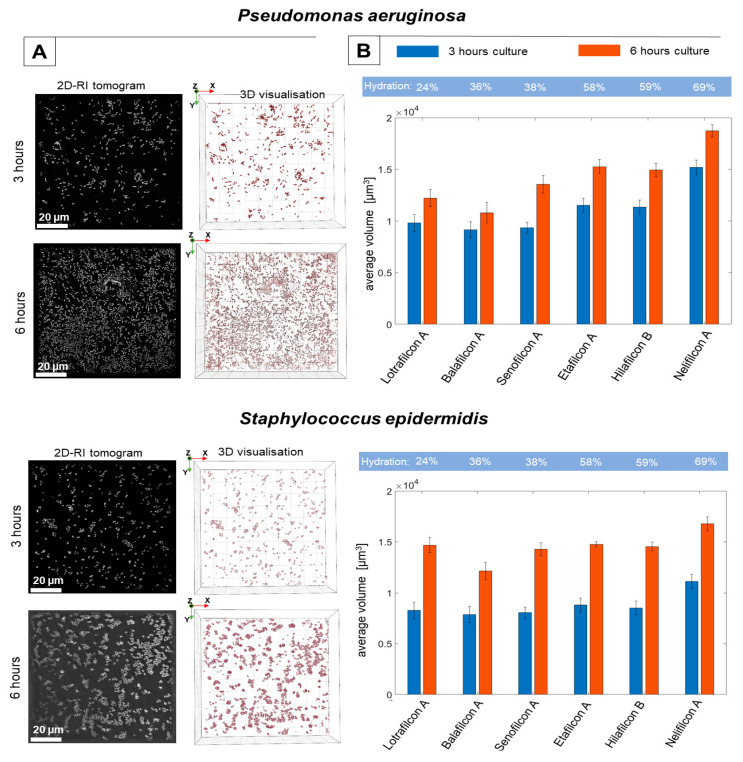
The results demonstrating the dynamics of the *P. aeruginosa* and *S. epidermidis* biofilm formation on the surface of different soft contact lens materials: (**A**) the exemplary 2D-RI tomograms and rendered 3D visualizations of biofilm on the Lotrafilcon A surface, (**B**) the changes of the average volume of the biofilms, depending on the hydration of the material and time (blue bars—3 h of biofilm cultivation, red bars—6 h of biofilm cultivation).

In the case of *P. aeruginosa* biofilms (see Figure 1C), the average RI of the biofilm depends on the hydration of the material. For 3 h of biofilm cultivation, it was shown that the lowest average RI was obtained for the Lotrafilcon A (silicone hydrogel) material, with the lowest hydration being equal to 24%. This material contains the highest levels of silicone, which reduces the porosity of the material, thereby reducing the surface roughness and increasing its hydrophobicity. This can create uncomfortable conditions for bacteria attachment and biofilm development. For other materials, the average RI slightly increases, with an increasing hydration of the material. In case of the 6 h cultivation of biofilms, a similar tendency was observed. However, the average RI values of the biofilms were lower than those obtained by the cultivation of the biofilms for 3 h, particularly for the Lotrafilcon A material. In the context of the results presented previously, this decrease in the average RI value may indicate that the population of dying cells begins to predominate in the biofilm (see Figure 2B) after 6 h of cultivation, or that the lack of any nutrients induced the process of cell death. Furthermore, as the hydration of the examined material increases, the differences in the average RI values of biofilms on different materials decrease. Therefore, the low hydration, roughness, and high hydrophobicity of the soft contact lens material may affect the viability of the bacterial cells, which may be indicated by the changes in the average RI.

For the 3 h cultivation of the biofilms of *S. epidermidis* (see Figure 1D), the hydration of the material on which the biofilm is formed was not observed, and significant changes in the average RI value were not observed. For the 6 h biofilm cultivations, a similar trend occurred as for *P. aeruginosa* biofilms; however, a significant decrease in the average RI value was only observed for the Lotrafilcon A material, with the lowest hydration of all the tested soft contact lens materials, suggesting that such low hydration may affect the viability of bacterial cells, since higher hydration levels lead to a higher average RI of biofilms.

It should also be pointed out that the average RI of the bacteria cells and the range of its changes related to the physiological processes depends on the medium in which they are found or on which they are immobilized [28]. This should therefore be considered when comparing the RI data across the bacteria being tested.

### 2.2. RI-Based Quantitative Study of the Dynamics of Biofilm Formation on the Surface of Soft Contact Lens Materials

The dynamic of bacterial biofilm formation depends on different factors, including the morphology of bacterial cells (presence of flagella and pili), the cell’s motility, roughness/smoothness, and the hydrophobicity/hydrophilicity of the surface, etc. For the examination of the biofilm development of the surface of the soft contact lens materials, the DHT system was used in our study. The obtained results are presented in Figure 2.

Based on the reconstructed 3D-RI tomograms, it was possible to digitally stain the bacterial cells, which permanently attached to the surface due to their specific RI-values. The analysis of the 2D-RI tomograms and 3D visualizations (see Figure 2A) have shown that, on the surface of examined materials, in the case of the bacteria species *P. aeruginosa* and *S. epidermidis*, it is possible to distinguish the cells clusters that indicate biofilm formation. The presence of these characteristic clusters increases over time (after 3 and 6 h of cultivation), which suggests a progressive development of the spatial structures of the biofilm across both the investigated bacteria species.

In comparison to *S. epidermidis*, the outer cell structures of *P. aeruginosa* exhibit a significantly higher number of long pili (or fimbriae) (however, in *S. epidermidis* cells isolated from new-born infants, short pilus-like structures are observed [29]), which are primarily responsible for bacterial motility, adhesion, and the effectiveness of the bacterial cell attachment to the surface. Therefore, these morphological differences promote the more effective attachment of *P. aeruginosa* cells to the surface of each material studied, which may explain the results obtained for the initial 3 h cultivation of biofilms.

In our experiments, the varying kinds of soft contact lens materials (silicone-hydrogels and hydrogels) with different hydrations and porosities were examined. Based on the reconstructed RI-data related to the biofilm’s structures, it was possible to determine the volume of biofilms formed on the surface of the examined soft contact lens materials. Therefore, it was possible to quantitatively analyze the structure and biomass of the biofilms directly on the examined surfaces. The obtained results (see Figure 2B) demonstrated that the highest, initial average volume (after 3 h of bacteria cultivation) was achieved by the *P. aeruginosa* species for each type of material. In the case of the *S. epidermidis*, the initial average volume was lower for each material, which can be related directly to the morphology of bacterial cells.

In the case of the *S. epidermidis,* there are no significant changes in the average volume of biofilms developed on the different types of materials. However, in case of the *P. aeruginosa*, the average volume of the biofilms increases as the hydration of the material increases. This effect may be directly associated with the structural differences between the materials (hydrogels and silicone hydrogels) studied. Hydrogels contain polymer networks with numerous microscopic pores, rendering them more hydrophilic and with a higher water content [30,31]. In addition, the pore size increases as the water content of the material rises. It has been shown that in the case of water membranes [32], high hydrophilicity and porosity of the material can promote fouling or biofouling, which is defined as the accumulation of organic and inorganic matter on the surface of a material, due to a physical or chemical interaction between the contaminant and the material. In addition, high hydrophilicity increases the water permeability of the material, which can promote a more effective attachment of bacterial cells to the material surface. To counteract this, it is usually necessary to reduce the hydrophilicity of the material (achieving superhydrophobicity) and its surface porosity/roughness [33]. This is why silicone hydrogel contact lenses are used. Made from a polydimethylsiloxane, silicone hydrogels also contain silicone, which tends to attract water less than hydrogel materials, making them more hydrophobic. As a result, the structure of the polymer network in silicone hydrogel materials is more compact and less porous [31,34], which limits the biofouling, the attachment of the bacteria cells to the surface, and bacterial biofilm formation. The obtained results suggest that the higher porosity, water content, and hydrophilicity of hydrogels may promote attachment and colonization of the surface of the contact lens materials by cells containing long pili, such as *P. aeruginosa*. This tendency was not as prominent for *S. epidermidis*, where the type of material did not affect the average volume of the biofilm; only for the material with the highest hydration (Nelifilcon A) was the significant increase in the biofilm volume observed.

Furthermore, the time-dependent differences (exhibited by both the 3 and 6 h of cultivation) of the average volume of biofilms demonstrate that the dynamics of biofilm formation are higher in the case of *S. epidermidis* than in the case of *P. aeruginosa*. After 6 h of cultivation, the average volume of the biofilms of *S. epidermidis* increases significantly. The average volume of the biofilms of *P. aeruginosa* is still higher than that of *S. epidermidis* for the more porous and hydrated hydrogels. Contrastingly, another study found that after 24 h of cultivation, the biofilm biomass of *P. aeruginosa* on the surface of soft contact lenses was higher than that of *S. epidermidis* [35,36]. However, in this case, different stains of the same bacteria species were investigated. This discrepancy can be explained by the significantly shorter duration of biofilm cultivation (6 h versus 24 h). Furthermore, based on the RI data, it was possible to perform the analysis of the viability of bacteria cells (see Section 3.1) directly on the examined surfaces. As the changes in the mean RI for *P. aeruginosa* cells are much closer to the range of RI values corresponding to the dying cells of these species than for *S. epidermidis* cells, the viability of these cells can also be expected to decrease over time, which may lead to a more rapid decrease in both the volume and biomass of the *P. aeruginosa* biofilm when compared to the *S. epidermidis* biofilm. This effect may explain the larger increase in biofilm volume between the 3 and 6 h cultivations on the contact lens surface in the case of the *S. epidermidis* species.

### 2.3. RI-Based Characterisation of Bacterial Biofilm Formation on the Surface of Soft Contact Lens Materials, Both Untreated and Treated with Selected Pharmaceutical Agents

**Figure 3:**
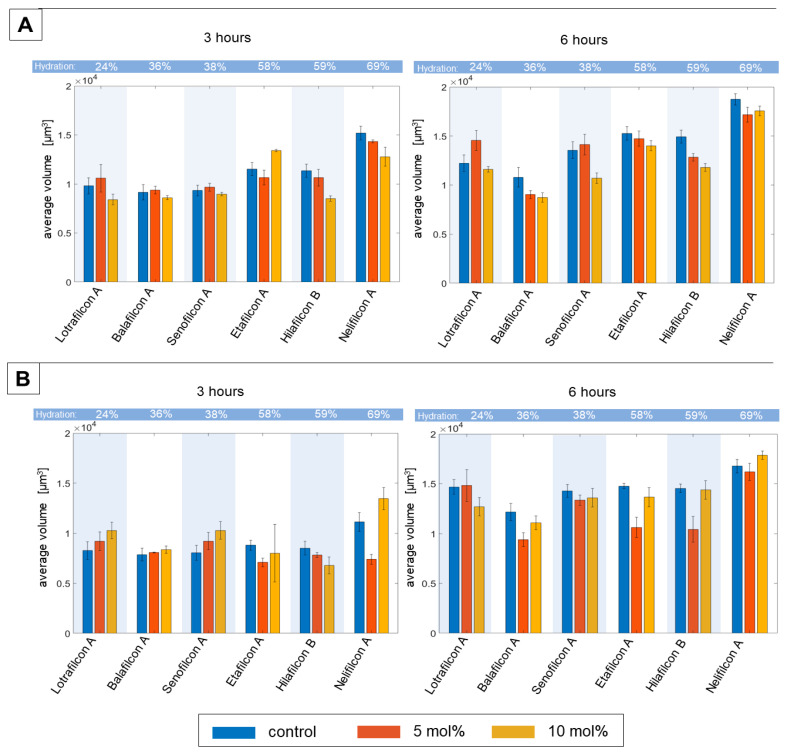
The time-dependent (3 and 6 h of biofilm cultivation) changes of the average volume of *P. aeruginosa* (**A**) and *S. epidermidis* (**B**) biofilms on the surface of six soft contact lens materials: untreated/control (blue bars) and treated by used pharmaceutical agent in two concentrations: 5 mol% (red bars) and 10 mol% (yellow bars) concentrations.

Various types of pharmaceuticals are used in the form of drops to treat specific eye diseases. However, very little research has been conducted on the possibility of applying pharmacological agents directly during lens wear [36,37,38,39]. In addition, there are few reports of alternative, rapid, and quantitative methods assessing the effect of drug application on the development of bacterial biofilms directly on the contact lens surface. Therefore, the subsequent stage of our research aimed to investigate the potential use of the DHT technique in characterizing the impact of an experimental glaucoma drug on the formation of bacterial biofilms on the surface of contact lenses. As in the previous case, the average RI and volume of the *P. aeruginosa* and *S. epidermidis* biofilms on the surface of three silicone hydrogels and three hydrogels was determined from the RI data that was obtained with DHT. The results are shown in Table 1 and Figure 3.

To examine the possible influence of the pharmaceutical used on the biofilms, the analysis of the difference in ΔRI between the average RI of biofilms on treated and untreated materials was performed (see Table 1). This examination was performed for two various times of biofilm cultivation (both 3 and 6 h) and two concentrations of the active agent (5 mol% and 10 mol%).

In this case, it was assumed that a value of ΔRI equal to zero or higher indicates no change in the cell viability or accumulation of the drug used, whereas a decrease in ΔRI may indicate a loss of cell viability, the initiation of cell death, or potential antibacterial activity. The obtained results highlight that in considering the standard deviation, there are no statistically significant changes between the treated/untreated samples, regardless of the concentration used. One can observe a trend where the average RI of 6 h cultivations of biofilms is lower (ΔRI < 0) when compared to biofilms on untreated materials (controls), suggesting a potential decrease in cell viability, particularly for *S. epidermidis*. However, it is worth noting that the high standard deviation of ΔRI may influence these observations.

More conclusive results were obtained for the changes in the average volume of the biofilms cultured on treated and untreated materials. In the case of *P. aeruginosa*, the changes in the average volume of the biofilm after 3 h of cultivation (see Figure 3A) with the pharmaceutical agent indicate that, for most types of materials, the volume was lower or at least comparable to the control sample (biofilms on the surface of untreated materials).

Considering the standard deviation, it can be concluded that the concentration of the pharmaceutical agent used did not affect the average volume of the biofilm. This was similar to or slightly lower than the average volume of the control sample. Only in the case of Etafilcon A did the average volume of the biofilm significantly increase with the increasing concentration of the active agent. After 6 h of biofilm cultivation, only for the Lotrafilcon A material was an increase in the average volume of the *P. aeruginosa* biofilm at lower concentrations observed. For most of the materials tested, the average volume of the biofilm was comparable to or smaller than the average volume of the control/untreated samples. In conclusion, the use of the pharmacological agent applied at two different concentrations does not significantly increase the development of the *P. aeruginosa* biofilm. Additionally, a comparable trend of an increasing average volume with heightened hydration of the material was observed at lower concentrations, similar to that seen in the control samples. At higher drug concentrations, this trend continued, except for in the Etafilcon A and Hilafilcon B materials. These results suggest that the hydration of the material is an important factor, which should be taken into consideration in such experiments.

In the case of *S. epidermidis*, considering the standard deviation, the changes in the average volume of the biofilm after 3 h of cultivation (see Figure 3B) were not relatively significant for most materials when compared to the control samples. For two silicone hydrogels (Lotrafilcon A and Senofilcon A) and for the hydrogel Nelifilcon A, the average volume was slightly higher than in the control sample. A probable increase in the average volume of the biofilm concerning the concentration of the active agent used can only be inferred for the Nelifilcon A material. After 6 h of cultivation, the average volume of the *S. epidermidis* biofilm was generally comparable to or smaller than that of the biofilm formed on the control sample. Therefore, as in the case of *S. epidermidis* also, it can be concluded that the pharmaceutical agent used did not significantly affect the biofilm formation on the surface of treated contact lens materials when compared to control samples.

The obtained results related to the changes in the average volume of the biofilms indicate a limited influence of the pharmaceutical agent used, which may explain the lack of significant changes in the ΔRI of the biofilms on treated/untreated materials.

## 3. Materials and Methods

**Figure 4:**
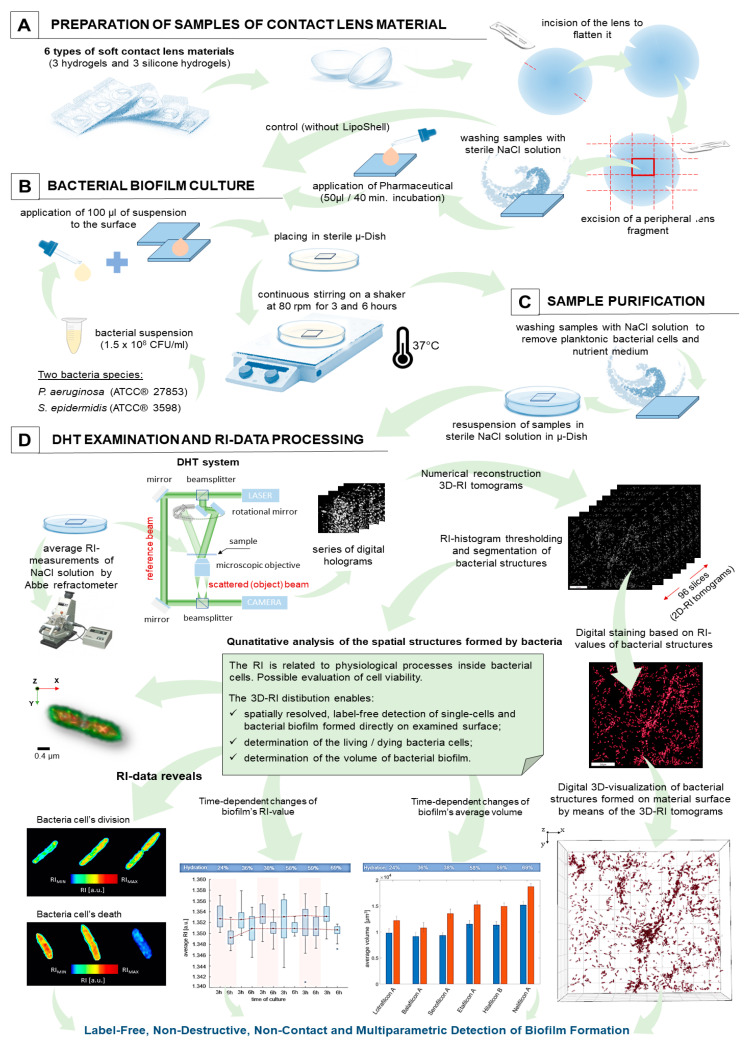
The schema of the sample’s preparation, measurements, and RI data analysis (scale bar 100 µm, microscope objective 60×).

The aim of this study was to characterize the dynamics of biofilm formation on the surface of the most common type of soft contact lens material, as well as to verify the potential use of digital holographic tomography (DHT) to perform this task in a truly label-free, non-contact, and non-destructive manner. The methodology of the procedures utilized is shown in Figure 4.

### 3.1. Contact Lens Samples

In this study, six types of soft contact lens materials from various manufacturers were selected, representing two of the most popular material classes: silicone hydrogel (Lotrafilcon A, Senofilcon A, Balafilcon A) and hydrogel (Etafilcon A, Hilafilcon B, Nelifilcon A). These had different hydration levels, wearing schedules, and oxygen permeabilities (see Table 2), ensuring the study covered a wide range of material properties.

Initially, a radial incision was made with a scalpel blade in order to flatten each of the tested lens samples (see Figure 4A). Then, for the direct observation and quantitative analysis of bacterial biofilm development directly on the surface of the contact lens material, 0.5 × 0.5 cm flat material samples were prepared for each lens material type. As the contact lenses were originally placed in storage solutions, the samples were next rinsed with a sterile 0.9% NaCl solution (Polpharma, Starograd Gdanski, Poland).

### 3.2. Pharmacutical Agent

In our study, we investigated the effect of novel liposomal latanoprost formulations without preservatives on the dynamics of biofilm formation on soft contact lens materials. Two liposomal samples were prepared by Liposhell technology [40], containing two concentrations of the Latanoprost (5 mol% and 10 mol%). The organic phase of the formulation was prepared by mixing an appropriate amount of purified phospholipids (Phospholipon 90 G, Lipoid GmbH, Ludwigshafen am Rhein, Germany), propylene glycol (BASF SE, Ludwigshafen, Germany), oleylamine (Sigma Aldrich, Saint Louis, MO, USA), and latanoprost (Angene Chemical, Telangana, India). Next, the extrusion process was conducted against the aqueous phase containing 0.9% NaCl through polycarbonate 100 nm filters (Whatmann, Nucleopore, Maidstone, UK). After preparation, the size distribution and zeta potential of liposomes in the final formulations were determined, along with the final chemical composition of the liposomes (Figures S1–S3 and Tables S1 and S2), which is described in detail in the Supplementary Materials. After the preparation of the samples as outlined above, 50 µL of the liposomal drops with two different concentrations (5 mol%, 10 mol%) of latanoprost as active ingredient were applied to the surface of each material type and allowed to remain for 40 min (see Figure 4A). This method of drug delivery has been selected due to its similarity to the administration of conjunctival drops.

### 3.3. Bacteria Samples Preparation

In our study, two reference strains of *S. epidermidis* (PCM 2532/ATCC^®^ 3598) and *P. aeruginosa* (PCM 2058/ATCC^®^ 27853) obtained from the Polish Collection of Microorganisms were used as model microorganisms capable of forming biofilms. Initially, a bacterial suspension was prepared from a 24 h cultivation at a density of 0.5 McFarland (1.5 × 10^8^ CFU/mL). Each sample of contact lens material was placed in a sterile dish (µ-Dish 35 low, IBIDI GmbH, Gräfelfing, Germany) in 3 mL of Tryptic Soy Agar (BTL, Warsaw, Poland), inoculated with 100 µL of each bacteria species (see Figure 4B). Following this, the whole sample (surface samples, nutrient medium, and bacteria cells) was kept under continuous stirring in a shaker (HLC Heating-ThermoMixer MHR 11, Digital Biomedical Imaging Systems AG, Pforzheim, Germany) at 80 rpm at 37 °C. The biofilm-covered samples of the contact lens materials (treated/untreated by the pharmaceutical agent) were disassembled after 3 and 6 h of cultivation, respectively, and the excess nutrient medium was washed off with a sterile 0.9% NaCl solution (Polpharma, Poland) (see Figure 4C). Then, the µ-dishes with the samples were supplemented with 2 mL of 0.9% NaCl solution, and the DHT examination was performed. Five biofilm samples (treated) and five reference samples (untreated) were prepared for each cultivation time, each contact lens material, and each active agent concentration.

### 3.4. Biofilm Examination by DHT

The DHT system (3D Cell Explorer, Nanolive, Tocholenz, Switzerland) was used to assess the dynamics of bacterial biofilm formation on the surface of six types of contact lens material (three silicone hydrogels, three hydrogels), untreated/treated with LipoShell (see Figure 4D). DHT is based on the configuration of the Mach–Zehnder interferometer. Therefore, the presence of the bacterial biofilm on the surface of the tested material will affect the phase difference between the two interfering beams used for digital hologram registration. The illumination of the sample by the beam at different angles of incidence allows for the registration of a series of digital holograms, from which the 3D refractive index (RI) distribution (3D RI tomogram) can be reconstructed numerically. Each 3D-RI tomogram contains 96 slices (2D-RI) tomograms. In total, 1159 3D-RI tomograms were recorded, of which more than 550 3D-RI tomograms per bacteria species were recorded for six types of material (treated/untreated), for two various biofilm cultivation times, and for two different concentrations of the active agent. After thresholding, the RI-histogram of each 3D-RI tomogram, it became possible to perform the segmentation of bacterial biofilm structures by extracting the regions occupied by bacteria cells. This enabled the determination of the average RI-values of biofilms, based on the maximum refractive index projections and their average volume, based on the number of the voxels corresponding to the bacterial structures. The RI data processing was performed using Matlab software (Matlab 2022b, Mathworks, Natick, MA, USA). The numerical reconstruction of the 3D-RI tomograms and the digital staining of the biofilms based on RI-values was performed using STEVE software (v. 1.6.3496, Nanolive, Ecublens, Switzerland). To numerically reconstruct the digital holograms, the reference RI measurement of the NaCl solution in which the samples were immersed (materials samples with bacteria) was performed using the Abbe refractometer (NAR-2T, minimum scale: 0.001, ATAGO Co. Ltd., Tokyo Japan) at 20 °C.

## 4. Conclusions

This study highlights the significant capabilities of DHT in deepening the knowledge of biofilm dynamics on soft contact lens materials. DHT is proven to be a powerful tool for monitoring the refractive index (RI) changes, a metric that is directly related to the density and chemical composition of the samples under investigation. Variations in the RI serve as valuable indicators of ongoing physiological processes that range from basic functions, such as cell division, to cell death. Time-lapse observations also reveal the complexity of biofilms, where cells undergo different stages of division, resulting in fluctuating RI values. The study demonstrates that the morphology of the bacterial cells plays a key role in this dynamic. *S. epidermidis* cells, with their smaller size and higher density, exhibit significantly higher average RI values when compared to *P. aeruginosa* cells. Moreover, it was shown that the cellular RI initially reflects the characteristics of living cells, but then undergoes transformations as the cells enlarge, leading to a decrease in the density and RI. Finally, these cells experience a decrease in size and RI as they approach death. The RI in *S. epidermidis* cells was observed to decrease more significantly with time than in its *P. aeruginosa* counterparts; this is primarily due to the differences in cell structure and size. Therefore, RI may become a valuable marker of bacterial cell viability, providing direct insight into its dynamic processes.

The analysis of biofilm dynamics does not stop at the cellular level but looks at the entire biofilm community. This investigation revealed that the collective RI of the biofilm is largely dependent on the predominant population, which can change over time. Time-lapse studies show that material properties such as hydration, surface roughness, and hydrophobicity have a significant impact on cell viability. For example, *P. aeruginosa* biofilms have lower RI values when the material is less hydrated. With prolonged biofilm cultivation, these cultures show a potential shift towards a predominant population of dying cells. Interestingly, the differences in the average RI values decrease as the material becomes more hydrated. In contrast, in the case of the biofilms of *S. epidermidis,* the average RI remains stable for 3 h of cultivation, except in cases where the material has very low hydration, leading to significant decreases in the average RI, which indicates a negative impact on cell viability. Furthermore, the study acknowledges the multifaceted nature of biofilm dynamics by highlighting the influence of factors, such as bacterial cell morphology and motility, and surface properties, like hydrophilicity. The use of digital holographic tomography reveals distinct cell clusters that are indicative of ongoing biofilm formation. These clusters progressively intensify over time, highlighting the dynamic nature of both *P. aeruginosa* and *S. epidermidis* biofilms on soft contact lens materials.

In addition to understanding the basics of the dynamics of biofilm formation, this study expands its focus to the effect of an experimental glaucoma drug on the development of biofilms directly on contact lens surfaces. The results indicate that for *P. aeruginosa*, the active agent’s concentration has a minimal effect on the biofilm volume at 3 h of cultivation, with some treated materials reducing biofilm volume when compared to the control samples. After 6 h, only one material, Lotrafilcon A, showed a significant increase in biofilm volume at lower drug concentrations. This suggests that under most conditions, the used pharmaceutical agent does not significantly enhance the development of *P. aeruginosa* biofilms. However, material properties, particularly hydration, play an important role in these results. In the case of *S. epidermidis*, the influence of the drug on biofilm formation is similarly limited, with minor fluctuations observed after 3 h, and a subsequent decrease after 6 h. Overall, it is evident that the experimental drug has minimal effects on *S. epidermidis* biofilm formations on contact lens materials when compared to the control samples.

In conclusion, this study demonstrates the prospect of using digital holographic tomography to delve into the complex dynamics of biofilm development, investigating the basis of its growth and the various factors influencing its progression, in a quantitative and completely non-destructive, non-contact, and label-fee manner. It is worth noting that our findings can be used in the future to further study the potential development of bacterial biofilms on the surfaces of different types of transparent multifunctional natural and synthetic biomaterials (hard/soft) [41], the biofouling associated with the hydrophilicity/hydrophobicity, or the porosity of the materials or membranes studied, as well as the potential influence of the agents/pharmaceuticals or surface modifications on biofilm growth.

## 5. Patents

The novel liposomal Latanoprost formulation used in this study is patent pending (patent application No WIPO ST 10/C PL447175).

## Figures and Tables

**Table 1:** The changes in the difference between the average RI of biofilm cultivations on treated and untreated (control) soft contact lens materials for two different concentrations of pharmaceutical agents and two different times of biofilms cultivation (bold indicates negative ΔRI values, or a decrease in the RI, relatively to the RI of the control sample).

*Pseudomonas aeruginosa*			*Staphylococcus epidermidis*	
Material ^1^	ΔRI (stdRI) × 10^−3^ [a.u.]		ΔRI (stdRI) × 10^−3^ [a.u.]	
	3 h	6 h	3 h	6 h
Lotrafilcon A + 5 mol%	+0.01 (1.37)	**−0.71** (2.57)	**−0.38** (3.72)	**−0.35** (1.72)
Lotrafilcon A + 10 mol%	+0.01 (1.37)	**−0.71** (2.56)	**−1.22** (3.26)	**−0.44** (2.54)
Senofilcon A + 5 mol%	+0.57 (2.76)	**−0.51** (2.91)	**−0.41** (4.78)	**−1.86** (6.64)
Senofilcon A + 10 mol%	+0.77 (2.66)	**−0.84** (3.14)	**−0.63** (4.60)	**−0.52** (5.92)
Balafilcon A + 5 mol%	+0.12 (2.19)	**−0.25** (2.39)	+0.11 (5.13)	**−0.69** (2.87)
Balafilcon A + 10 mol%	+0.12 (2.39)	**−0.38** (2.22)	**−1.87** (5.66)	**−1.07** (2.49)
Etafilcon A + 5 mol%	**−0.05** (3.12)	+0.06 (2.33)	+3.54 (6.93)	**−0.73** (4.28)
Etafilcon A + 10 mol%	+0.30 (2.82)	+0.07 (2.44)	+1.76 (7.97)	**−0.62** (2.89)
Hilafilcon B + 5 mol%	**−0.72** (3.82)	**−0.06** (2.86)	**−0.09** (7.26)	**−1.34** (6.45)
Hilafilcon B + 10 mol%	**−0.78** (3.43)	**−0.09** (2.39)	+1.63 (7.35)	**−0.41** (3.72)
Nelifilcon A + 5 mol%	**−0.38** (1.57)	+0.10 (2.04)	**−1.12** (2.83)	**−2.41** (4.32)
Nelifilcon A + 10 mol%	**−0.35** (1.52)	**−0.17** (2.35)	**−1.68** (2.80)	**−0.92** (2.85)

^1^ Examined material treated with different concentrations of the pharmaceutical agent (5 mol%/10 mol%).

**Table 2:** The specification of the examined soft contact lenses (manufacturer data).

Material	Manufacturer	Wear Schedule	Dk ^1^ (fatt/Iso)	Hydration
Lotrafilcon A	Alcon(Geneve, Switzerland)	30 days	140	24%
Senofilcon A	Johnson & Johnson (Irvine, CA, USA)	14 days	103	38%
Balafilcon A	Valeant—Bausch & Lomb (Quebec, QC, Canada)	30 days	130	36%
Etafilcon A	Johnson & Johnson (Irvine, CA, USA)	1 day	33	58%
Hilafilcon B	Valeant—Bausch & Lomb (Quebec, QC, Canada)	14 days	19	59%
Nelifilcon A	Alcon (Geneve, Switzerland)	1 day	26	69%

^1^ Dk (oxygen permeability of the material) measured based on the Irving Fatt method, indicating the absolute amount of oxygen diffusing through the material.

## Data Availability

All data can be obtained from authors on request.

## Supplementary Materials

The supporting information can be downloaded at: https://www.mdpi.com/article/10.3390/ijms25052653/s1. Reference [42] is cited in the Supplementary Materials.
