# Supplementary material for: Quantifying the Dynamics of Bacterial Biofilm Formation on the Surface of Soft Contact Lens Materials Using Digital Holographic Tomography to Advance Biofilm Research
Source: Int J Mol Sci. 2024 Feb 24;25(5):2653. doi: 10.3390/ijms25052653 (PMC10932241; doi:10.3390/ijms25052653)
Supplement: Supplementary file 1 [file ijms-25-02653-s001.zip › ijms-2872464-supplementary.pdf]

Supplementary Materials

Article

Quantifying the Dynamics of Bacterial Biofilm Formation on the Surface of Soft Contact Lens Materials Using Digital Holographic Tomography to Advance Biofilm Research

Igor Buzalewicz [1,2,*], Aleksandra Kaczorowska [1,3], Wojciech Fijałkowski [4], Aleksandra Pietrowska [1], Anna Karolina Matczuk [5], Halina Podbielska [1], Alina Wieliczko [6], Wojciech Witkiewicz [2] and Natalia Jędruchniewicz [2]

1 Department of Biomedical Engineering, Faculty of Fundamental Problems of Technology, Wroclaw University of Science and Technology, 50-370 Wroclaw, Poland; aleksandra.kaczorowska@pwr.edu.pl (A.K.); aleksandra.pietrowska@pwr.edu.pl (A.P.); halina.podbielska@pwr.edu.pl (H.P.)
2 Research and Development Centre, Regional Specialist Hospital in Wroclaw, 73A H. M. Kamienskiego St., 51-124 Wroclaw, Poland; wojciech.witkiewicz@wssk.wroc.pl (W.W.); natalia.jedruchniewicz@wssk.wroc.pl (N.J.)
3 Laboratory of Cytobiochemistry, Faculty of Biotechnology, University of Wroclaw, 14a F. Joliot-Curie St., 50-383 Wroclaw, Poland
4 LipoTech Ltd., Liszki 536, 32-060 Liszki, Poland; wojtek.fijalkowski@lipid-systems.pl
5 Department of Pathology, Division of Microbiology, Faculty of Veterinary Medicine Wroclaw University of Environmental and Life Sciences, 31 C.K. Norwida St., 51-375 Wroclaw, Poland; anna.matczuk@upwr.edu.pl
6 Department of Epizootiology and Veterinary Administration with Clinic of Infectious Diseases, Wroclaw University of Environmental and Life Sciences, 45 Grunwaldzki Square, 50-366 Wroclaw, Poland; alina.wieliczko@upwr.edu.pl
* Correspondence: igor.buzalewicz@pwr.edu.pl

Four pages of supplementary materials including 3 supplementary figures (Fig.S1, Fig.S2, Fig.S3) and two supplementary tables (Table S1, Table S2).

S1. The composition of the studied formulations

The detailed information about the composition of the novel liposomal formulations of latanoprost studied and its characteristics are indicated in **Table S1**. The formulations of two concentrations (5mol%/10mol% with respect to the phospholipid content) of Lantanoprost (LAT) were examined by dynamic light scattering size distribution and Zeta potential methods, in PBS buffer pH 7.4 and conductivity 0.4 mS/cm.

Table S1. The composition of the studied formulations with the measured parameters of liposomes.

Composition of the formulation	Z- ave	PdI	PdI	Zeta potential
	[nm]	[-]	[nm]	[mV]
PC12.72 mM /PG/OE5mol%/LAT5mol%	125,1	0,052	28,46	18.0
PC12.72mM/PG/OE5mol%/LAT10mol%	125,6	0,059	30,22	19.8

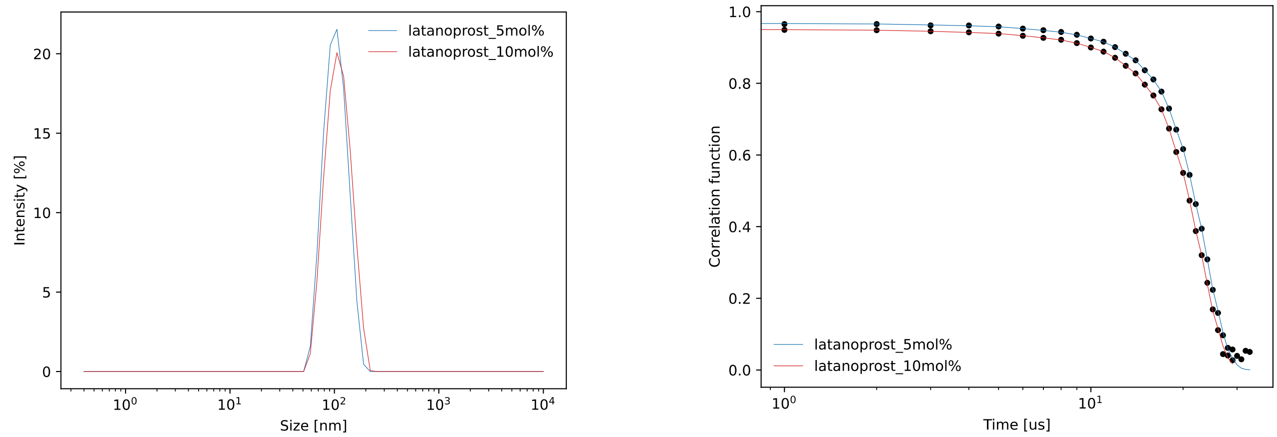

A B

Figure S1. Size distribution histogram (A) with corresponding correlation functions (B) for both liposomal Latanoprost formulations showing the uniform distribution of the liposomes.

S2. Organic phase extraction

To correctly determine the content of the formulation, a modified method of extracting the organic phase from the solution developed by E. G. Biligh and W. J. Dyer[1] was used. To 1 ml of the prepared formulation, 1 ml of methanol and 2 ml of chloroform were added. The whole mixture was vigorously shaken and centrifuged at 3000 RPM for 10 minutes. The separated organic fraction was then collected and analyzed directly on HPLC with ELSD and UV-VIS detection with recovery rate of 84,98%.

[1] Bligh Eg, Dyer Wj. A Rapid Method Of Total Lipid Extraction And Purification. Can J Biochem Physiol. 1959 Aug;37(8):911-7. Doi: 10.1139/O59-099. Pmid: 13671378.

S3. HPLC analysis

High-performance liquid chromatography (HPLC) separation of the extracted organic phase was used for the latanoprost and phospholipid content determination. The method utilized time-varying gradient of two phases given in **Table S2.**

Table S2. HPLC gradient for the separation of latanoprost and phospholipids.

Time	Phase A	Phase B	Flow
[min.]	[%]	[%]	[ml min^{-1}]
0	100	0	1
3	95	5	1
5	85	15	1
8,5	60	40	1
15	0	100	1
17,5	0	100	1
17,6	100	0	1
19	100	0	1
20	100	0	2
22,5	100	0	2
24	100	0	1

A mixture of hexane-2-propanol 83:17 (v:v) was used as phase 'A', while phase 'B' was a mixture of water-isopropanol 11:68 (v:v). The analysis was performed using a Knauer HPLC system (Knauer, Germany) and Lichrospherer 100-5 Diol column under 55°C. Measurements were carried using ELSD (Alltech, USA) and UV-VIS (Knauer, Germany) flow detector at 210 nm wavelength. Sample chromatograms are presented on the **Figure S2**.

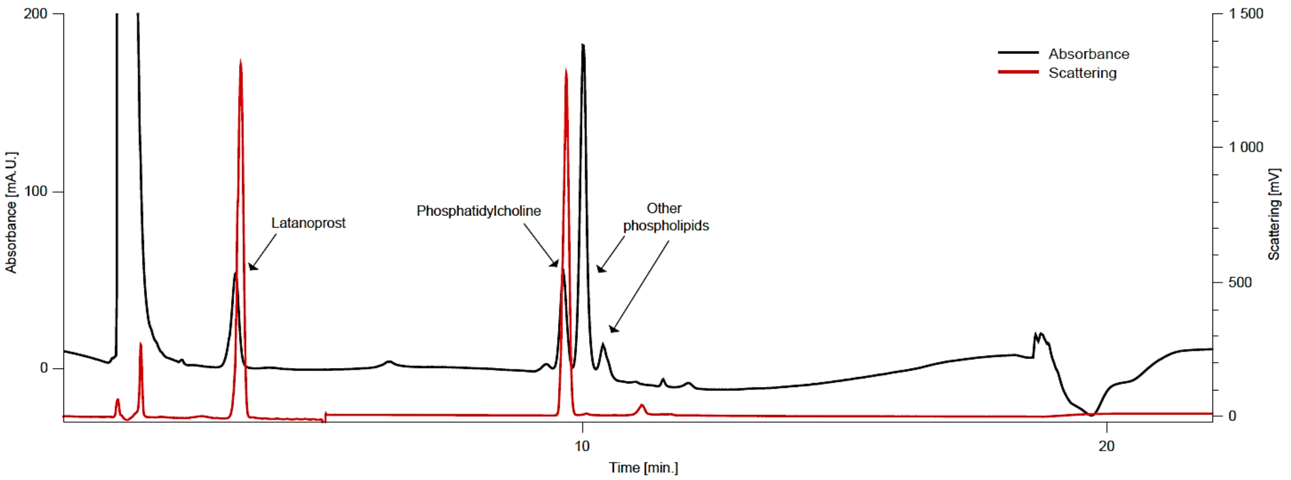

Figure S2. Chromatograms of the liposomal formulations after extraction with separated latanoprost and phospholipids.

Figure **S3** presents the linearity of the method.

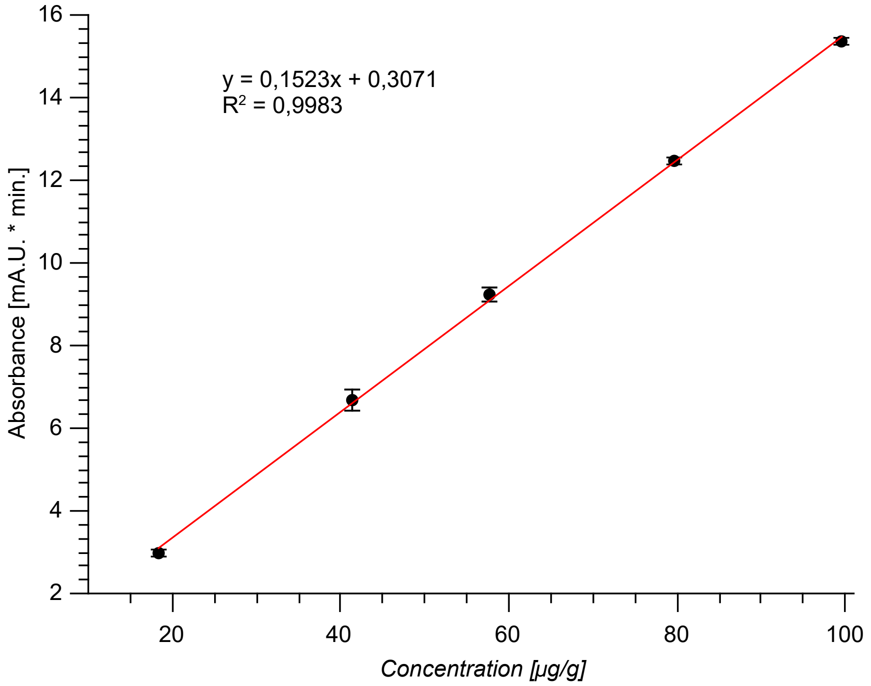

Figure S3. The linear relation between the latanoprost concentration and AUC of the absorbance at 210 nm peak for latanoprost standard.
